# P013: Prevalence rate of healthcare associated infection in emergency critical care for surgery, burns and neurology ward

**DOI:** 10.1186/2047-2994-2-S1-P13

**Published:** 2013-06-20

**Authors:** S Mehtar, JMV Namahoro

**Affiliations:** 1 Infection Prevention and Control, Stellenbosch University, Cape Town, South Africa

## Introduction

Many Healthcare Associated Infections (HAI) are related to the use of a medical device, especially invasive devices that buy-pass the normal barriers to entry of microorganisms into the body. HAI rates of CA-UTI, CLA-BSI and VAP are significantly greater among patients when a medical device is used

## Objectives

Determine Healthcare Associated infection risk with medical devices

To make recommendations for performance improvement

## Methods

Observation study of pilot study. Data was collected for a period of five weeks. Active surveillance by considering laboratory results and regular visits to patients in respiratory intensive care unit was a method used to assess patients at-risk of healthcare associated infection. The Structured audit sheet was used

## Results

The sample consisted of 92% patients received urinary catheter, 83% patients are exposed on the peripheral lines, 50% patients obtained central lines and 58% patients acquired endotracheal tube in situ. The mean length of stay was 4.04. The prevalence rate of HAI was 50% infections. The predominance of prevalnce rate was found for VAP by 36%, while incidence rate of CLA-BSI was 25% and incidence rate in CA-UTI with Urinary tract infection was 14%

## Conclusion

The HAI is very high in C1 Resuscitation ward. The improved IPC practice is required and further research is needed to identify the effect of medical devices with prevalence of infection in C1 Resuscitation ward

## Disclosure of interest

None declared
